# A Density Functional Theory (DFT) Study of the Acyl Migration Occurring during Lipase-Catalyzed Transesterifications

**DOI:** 10.3390/ijms20143438

**Published:** 2019-07-12

**Authors:** Jinyuan Mao, Zhenying Hu, Jiangning Hu, Xuemei Zhu, Hua Xiong

**Affiliations:** 1 State Key Laboratory of Food Science and Technology, Nanchang University, Jiangxi 330047, China; 2 School of Food Science and Technology, Dalian Polytechnic University, Dalian 116034, China

**Keywords:** acyl migration, density functional theory, lipase-catalyzed mechanism, structural triglycerides, QM

## Abstract

Acyl migration (AM) is the main side reaction in the large-scale, regio-specific lipase catalyzed production of structural triglycerides (STs). A detailed understanding of the mechanism of AM was obtained during the process of lipase-catalyzed schemes (LCSs), which play a vital role in improving the quality and total yield of STs. However, currently, the mechanism of AM remains controversial. Herein, the two mechanisms (non-catalyzed (NCM) and lipase-catalyzed (LCM)) of AM have been analyzed in detail by the density functional theory (DFT) at the molecular level. Based on the computational results, we concluded that the energy barrier of the rate-limiting step in the LCM was 18.8 kcal/mol, which is more in agreement with the available experimental value (17.8 kcal/mol), indicating that LCM could significantly accelerate the rate of AM, because it has an energy barrier ~2 times lower than that of the NCM. Interestingly, we also found that the catalytic triad (Asp-His-Ser) of the lipase and water could effectively drop the reaction barrier, which served as the general acid or base, or shuttle of the proton.

## 1. Introduction

Lipase, which belongs to the class of serine hydrolases (E.C. 3.1.1.3) [1], can catalyze the hydrolysis of oils and fats by cleaving long-chain triglycerides (TGs) in order to liberate fatty acids and glycerol [2]. A range of lipase-catalyzed schemes (ester-ester exchange, esterification, interesterification, transesterification, etc.) (LCSs) has been used for many years to improve the properties of synthetic structural TGs (STs) in the food industry [3]. LCSs, as the preferred means, have been extensively applied in the production of cocoa butter candidates, human milk fat substitutes, medium-long-medium-type STs, etc., contributing to its region-selectivity, effectivity, substrate specificity, mild reaction, and safety [3,4].

However, acyl migration (AM), occurring and observed in regio-specific STs, even the region-specific lipase, used as a catalyst, which is well known as a severe problem in synthesis because of its lower purity and generation of free fatty acids, monoglycerols (MGs), diglycerols (DGs), and TGs, seriously influences the quality and total yield of STs. Moreover, AM cannot be easily avoided as a side reaction during the process of LCSs [5,6].

Several aspects related to the possible induction of AM are summarized below. (1) The temperature and reaction time, which are recognized as thermodynamic factors, influence AM by the thermodynamic equilibrium. According to Arrhenius’ law, the rate of AM increases sharply with the increase of the reaction temperature and time [7]. A longer time is needed to reach equilibrium under a lower temperature, since the reaction time is a parameter related to the reaction temperature. Moreover, a long-time reaction leads to the accumulation of DG, resulting in a magnification of the AM [8]. (2) The water activity (defined as the ratio of the water vapor pressure of the food to the vapor pressure of pure water at the same temperature [9]) and solvent influence AM. A high water activity is not directly conducive to a high rate of AM. It probably affects the activation energy of the reaction by disturbing the electron distribution of the transition state (TS), thus shaping AM [10]. Besides, DG content yield depends on water activity, resulting in a different degree of AM [11]. In the case of a solvent, generally higher polar solvents (chloroform, acetone, etc.) would reduce the rate of the AM [12], but this does not always occur [13]. (3) The enzyme and support influence AM. The content of enzyme preparation is positively correlated with AM [14], and the support of enzymes (resins, silicas, etc.) induce AM as well [15,16]. (4) Other factors, such as the degree of branching, unsaturation, and the acyl group length of fatty acid in substrates affect AM by the equilibrium distribution of DG and MG. The more MG acyl groups that contain branched, unsaturated, and short-chain fatty acids, the faster the rate of AM [5,17].

In the past few years, some researchers have paid intensive attention to illustrating the mechanism of AM. One of the hypotheses, known as the non-catalyzed mechanism (NCM) of AM, has been proposed by the nucleophilic attack of electrons of the hydroxyl oxygen on the ester carbonyl carbon, resulting in an unstable five-membered cyclic intermediate [11,18]. Further works also deduced the existence of NCM in the presence of a ketal intermediary [19]. Additionally, it was confirmed, as an aside, that the primary hydroxyl oxygen was a better nucleophile than the secondary hydroxyl, and the acyl that shifted from a secondary hydroxyl to a primary hydroxyl was, therefore, preferred [5]. Additionally, some reports have also demonstrated that both the lipase load and lipase type could affect AM, and the AM of 1(3)-MG to 2-MG was a process involving with lipase. Taken together, it seemed that the NCM and lipase-catalyzed mechanism (LCM) of AM occurred simultaneously [20]. Even though many related factors and different proposed mechanisms of AM were discussed, the mechanism of AM is still uncertain due to the difficulty involved in capturing TS or its intermediate (IM) to sufficiently support the hypotheses. To address this controversy, it is important to provide insight into the mechanism of AM during the process of LCSs at the molecular level. Quantum chemical simulation has now drawn unprecedented attention due to its better explanation of the conclusions derived from experiments at the molecular level, and the cluster model, focusing on a small part of the enzyme around the active site, has also been an effective technique to explore mechanistic insights into enzyme-catalyzed reactions over the past 20 years [21].

In the present study, quantum mechanics (QM) simulations have been performed to explore two proposed mechanisms (NCM and LCM) in detail. The hybrid density functional theory (DFT), which has been successfully used to study the catalytic reaction mechanism [22,23]., was employed to clearly shed light on these two mechanisms of AM during the process of LCSs, based on the quantum chemical models. The aim of this paper was to obtain a deeper understanding of the mechanism of AM during the process of LCSs, from a molecular point of view.

## 2. Computational Models and Details

### 2.1. Quantum Chemical Models

The computational investigations into the non-catalyzed and lipase-catalyzed mechanisms of AM have been carried out, based on simplified models. The initial 3D structure of 1,2-diacetin, as the non-catalyzed model, was downloaded from a database (PubChem CID: 66021). A water molecule was then manually added to consider its influence on AM in the NCM. The starting model of LCM was built based on the crystal structure of TG lipase from *Rhizomucor Miehei* in a complex with an inhibitor compound, diethyl phosphonate inhibitor (Protein Data Bank code: 4TGL) [24]. The inhibitor compound was manually changed to the substrate of 1,2-diacetin to construct the enzyme-substrate complex model, (ESC) 1,2-diacetin-4TGL. In our study, only the residues that have an important role in the catalytic reaction and correct positioning of the substrate were considered. The side chain of these residues was maintained, and their truncation points were kept frozen, according to their X-ray structural position. All hydrogen atoms were added manually. The ESC consisted of 123 atoms, and the following groups were included: (1) the catalytic triad (Asp203-His257-Ser144) in the active site, where Asp203 was a stand for acetate moiety, and His257 and Ser144 were represented by methyl imidazole ring and ethanol, respectively; (2) modified Val205 and Tyr260, forming hydrogen bonds with the carboxyl Oδ1 and Oδ2 of Asp203, respectively; (3) the side chain of Leu145, Ser82, and Tyr28, consisting of an “oxyanion hole”, which was important for stabilizing a charged oxygen atom, produced in the TS [25]. Moreover, a water molecule, as a shuttle of the proton, was also manually put into the appropriate position of the ESC model in the reaction.

### 2.2. Computational Details

All structures were optimized by employing a nonlocal correlation functional B3LYP [26,27,28], with a 6-31G(d,p) basis set. Harmonic vibrational frequency calculations were conducted at the same level of theory to characterize the nature of the stationary points, i.e., a minimum or TS, and provide thermal corrections to the Gibbs free energy at 298.15 K. Following the intrinsic reaction coordinates (IRC), all transition states connected the corresponding local minima on the potential energy surface (PES) were verified. The higher accurate electronic energies were obtained from single-point calculations by employing the 6-311G++(2d,2p) basis set. The solvation effects from the missing protein environment were taken into account by the implicit solvent model, i.e., solute electron density (SMD) solvation model [29], with the dielectric constant of 3.0. The total Gibbs free energies of all stationary points were taken as the sum of the gas-phase single-point energies, thermal corrections, and the solvation free energy. All computations were carried out using the Gaussian 09 software package [30].

## 3. Results and Discussion

**Figure 1:**
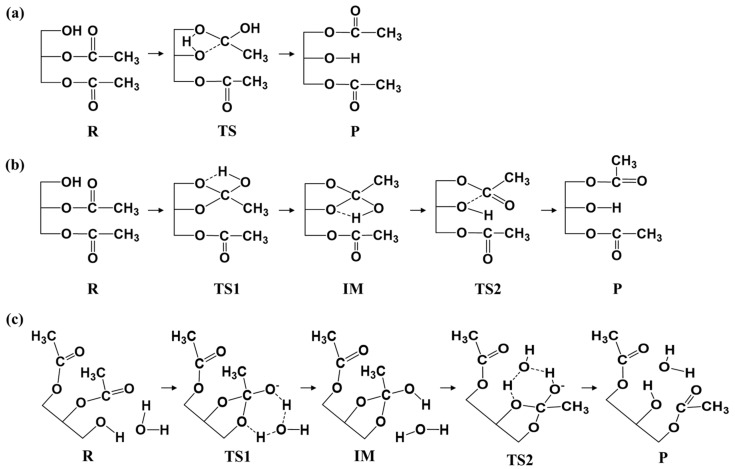
The possible mechanisms of acyl migration (AM) in the non-catalyzed mechanism (NCM) scheme: (**a**) concerted pathway; (**b**) stepwise pathway; (**c**) stepwise pathway with the aid of water.

**Figure 2:**
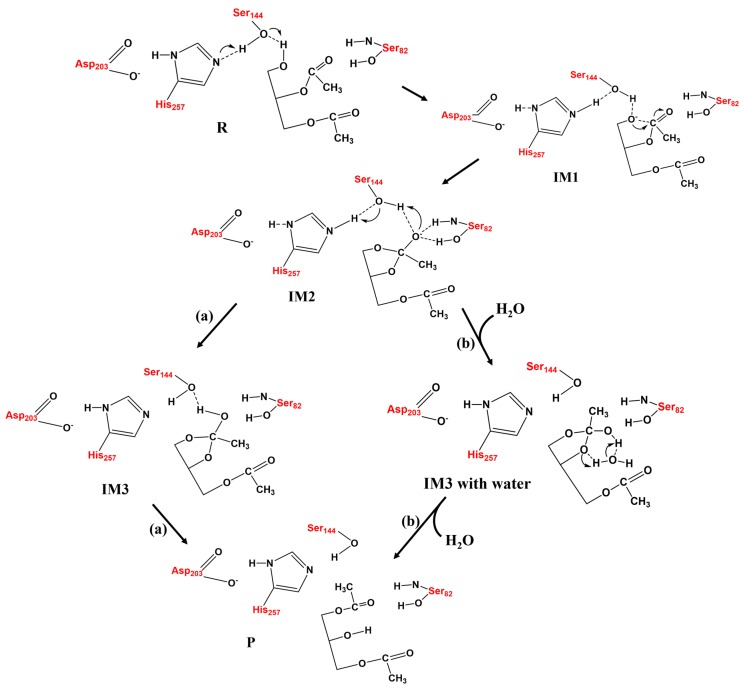
The possible mechanisms of acyl migration (AM) in the lipase-catalyzed mechanism (LCM) scheme: (**a**) without and (**b**) with the aid of water.

Two proposed mechanisms of NCM (Figure 1) and LCM (Figure 2) were detailed, based on the quantum chemical calculations, to address the controversies regarding the mechanism of AM during the process of LCSs.

### 3.1. NCM of AM

**Figure 3:**
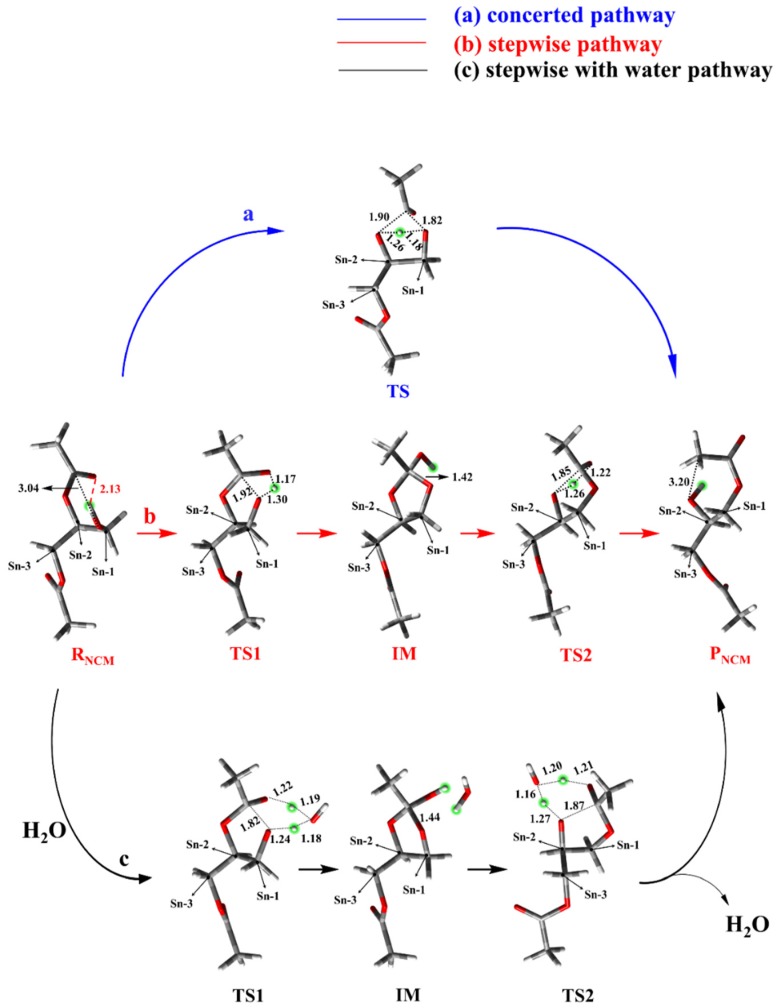
Optimized structures of the reactant (R_NCM_), transition state (TS), intermediate (IM), and product (P_NCM_) of NCM for the concerted pathway (**a**), stepwise pathway (**b**), and stepwise pathway with the aid of water (**c**).

**Figure 4:**
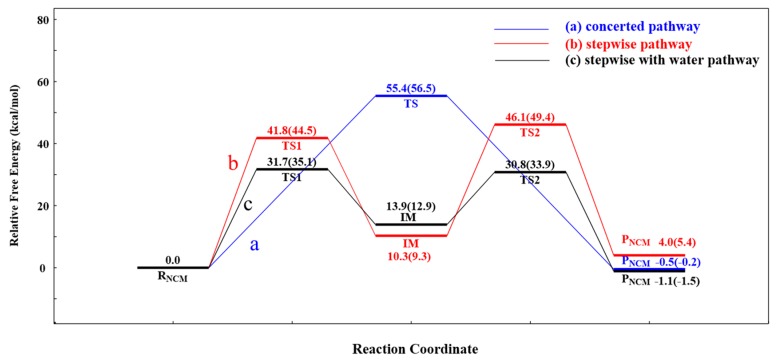
The calculated NCM of the AM free energy profiles: concerted pathway (**a**); stepwise pathway (**b**); stepwise pathway with the aid of water (**c**) in the solution with a thermal correction to the Gibbs free energy and in the gas phase (in parentheses), with zero-point energy (ZPE) correction. Relative free energies to the reactant are given in kcal/mol.

For NCM, the possible pathways, i.e., the concerted and stepwise pathways, without and with the aid of water (as shown in Figure 1), were proposed and explored. Figure 3 depicted the optimized structures of all stationary points on the PES of non-catalyzed AM, and the corresponding relative free energy of each structure is displayed in Figure 4. The most stable structure of 1,2-diacetin was regarded as the reactant (R_NCM_), where the sn-1 hydroxyl formed a hydrogen bond (2.13 Å) with the ester oxygen. In the concerted pathway, the transfer of the proton from the sn-1 hydroxyl to the ester oxygen of sn-2 and the cleavage of the sn-2 ester bond occurred synchronously. As shown in the TS of the concerted pathway, the proton of the sn-1 hydroxyl and the acyl group were located at the intermediate (IM) position, between the oxygen of sn-1 and sn-2, forming a strained four-membered ring. After the exchange between the proton of sn-1 and the acyl group of sn-2, the product (1,3-diacetin) was produced. The calculated activation-free energy was very high at 55.4 kcal/mol. The energy of 1,3-diacetin was almost equal to that of the reactant (1,2-diacetin). For the stepwise pathway, as shown in Figure 1 and Figure 3, the AM between sn-1 and sn-2 is completed in two steps: (1) the transfer of the proton from sn-1 to the carbonyl oxygen of sn-2, accompanied by the oxygen of the sn-1 hydroxyl nucleophilic attack on the sn-2 carbonyl carbon, forming a five-membered ring; and (2) the opening of the five-membered ring from the ester oxygen of sn-2, together with the transfer of the proton from the carbonyl oxygen to the ester oxygen of sn-2. As displayed in Figure 4, the activation energy of the rate-limiting step (the first step) in the stepwise pathway was 13.6 kcal/mol lower than that in the concerted pathway, suggesting that the AM of the stepwise pathway was more favorable. In addition, we also investigated the effect of the water molecule on the AM of the stepwise pathway to determine if water activity could accelerate AM [31]. The calculated results showed that the water molecule acted as a shuttle of the proton, helping to transfer the proton from sn-1 to the sn-2 of the substrate, 1,2-diacetin. The aid of one water molecule effectively lowered the activation energy barrier of the rate-limiting step in the stepwise pathway from about 10.l kcal/mol to 31.7 kcal/mol (as depicted in Figure 4). This effect can be easily explained. The participation of one water molecule led to the formation of a six-membered ring in the TSs of both steps, which are more energetically stable. Therefore, the water molecule, as both a base and a weak acid during AM, activated nucleophiles and electrophiles by hydrogen bonding, thus facilitating AM [32]. While the role of the water molecule dramatically lowered the activation energy barriers of AM, it was still so high that it was difficult for AM to occur under this simulated circumstance (298.15 K).

### 3.2. LCM of AM

**Figure 5:**
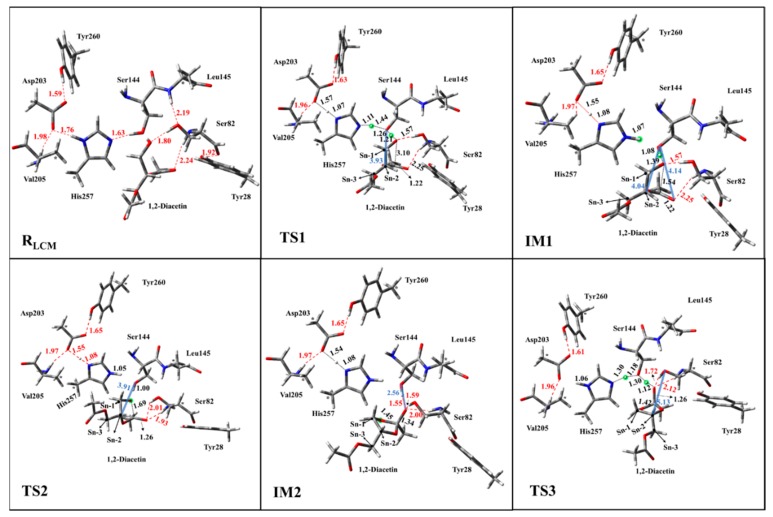
Optimized structures of the reactant (R_LCM_), transition state (TS1, TS2, TS3), intermediate (IM1, IM2) of the enzyme-substrate complex in the LCM of the AM. Red dashed lines represent the hydrogen bond. The hydrogen bond distances are represented by the red dashed line, the distance between substrate and catalytic triad are marked as the blue solid line, all distances are given in angstroms, and the asterisks (*) stand for the fix atoms during geometry optimization.

As the AM through NCM was extremely slow at room temperature due to an overwhelming energy barrier, the alternative mechanism, LCM, was explored as well. The initial reactant (R_LCM_) of LCM, represented by the active site of lipase with the substrate (1,2-diacetin), was restrictively optimized and is displayed in Figure 5. Hydrogen bond networks and the corresponding distances, formed between the residues of active site, can obviously be observed in Figure 5. It can also be observed that Ser82 and Ser144 provided the hydrogen bond interactions to stabilize the substrate. It is worth noting that the formed chain of the hydrogen bond, between Asp203-His257-Ser144 and the substrate, 1,2-diacetin, could favor the transfer of the proton. For LCM, the proposed pathway could be divided into four elementary steps.

**Figure 6:**
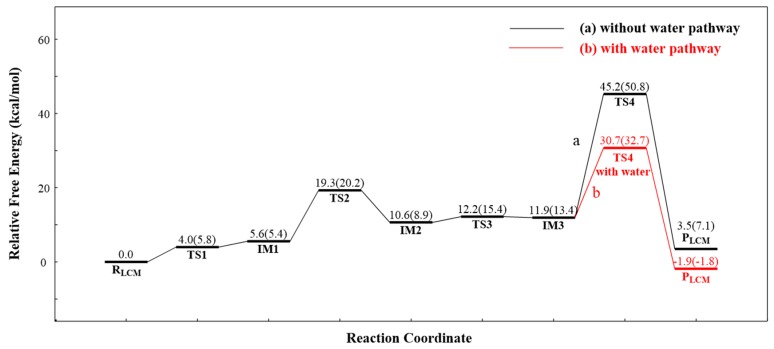
The calculated LCM of the AM free energy profiles in the solution and in the gas phase (in parentheses), without (black line) and with (red line) the aid of water. Relative energies with respect to the reactant are given in kcal/mol.

The double-proton transfer step: The deprotonation of 1,2-diacetin, with the help of the catalytic triad, was the first step in the pathway of LCM. The structures of TS1 and IM1 of the double-proton transfer are displayed in Figure 5. As seen from Figure 5-TS1, His257 deprotonated Ser144, while Ser144 captured the hydroxyl proton on the sn-1 of the substrate, 1,2-diacetin. The double-proton transfer was concerted but not completely synchronous, and the extraction of the proton by His257 from Ser144 occurred at a later stage. The corresponding distances marked in intermediate 1 (IM1) verified that 1,2-diacetin was successfully deprotonated by Ser144. Interestingly, compared with RLCM, the hydrogen bond between Asp203 and His257 seemed to become a “short-strong hydrogen bond” [33,34,35], which favored the lowering of the energy barrier of the double-proton transfer and stabilization of the positive charge of His257, formed by abstracting the proton from Ser144. Dynamically, the double-proton transfer step easily occurred due to the very low energy barrier (4.0 kcal/mol), but it was endothermic by 5.6 kcal/mol (Figure 6, R_(LCM)_-IM1). It can be noted that the reaction likely occurred without an energy barrier, since the IM1 was slightly higher than that of the TS1. This is a common result of using single-point calculations or empirical corrections on potential energy surfaces (PESs), which can be avoided at a higher level of theory calculation, without the inclusion of the environment’s electrostatic charge [36].

**Figure 7:**
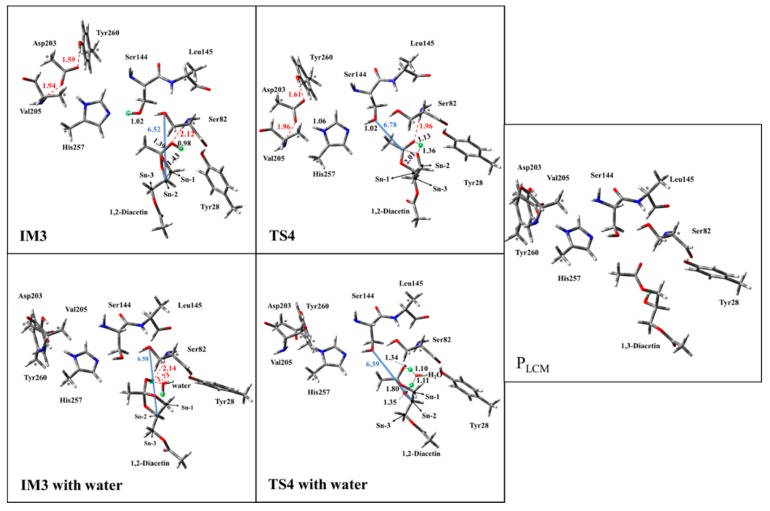
Optimized structures of the transition state (TS4, TS4 with water), intermediate (IM3, IM3 with water), and product (P_LCM_) of the enzyme-substrate complex in the LCM of the AM. Red dashed lines represent the hydrogen bond. The hydrogen bond distances are represented by the red dashed line, the distance between substrate and catalytic triad are marked as the blue solid line, all distances are given in angstroms, and the asterisks (*) stand for the fix atoms during geometry optimization.

The oxyanion on the sn-1 of the 1,2-diacetin nucleophilic attack on the sn-2 carbonyl carbon: After the deprotonation of 1,2-diacetin, the hydroxyl oxygen of sn-1 gained a strong nucleophilicity. The second step involved the oxyanion on the sn-1 of 1,2-diacetin nucleophilic attacking the sn-2 carbonyl carbon, resulting in a cyclic five-membered ring, IM2 (Figure 5), and a corresponding optimized structure of TS2 is depicted in Figure 7. Following the nucleophilic attack, the C=O double bond of carbonyl with 1.22 Å, shown in Figure 3, was gradually elongated to a C-O single bond with 1.34 Å (Figure 3), and the newly formed oxyanion was stabilized by Ser144 and Ser82 residues with the hydrogen bond interaction. We also note that the re-orientation of the substrate, 1,2-diacetin, occurred spontaneously, which may be due to the extraction of the proton from Ser144 in the next step (4.14 Å to 2.56 Å in Figure 5). The present nucleophilic attack step was endothermic (+5.0 kcal/mol), even though it needed to overcome the activation-free energy barrier of 13.7 kcal/mol.

Double-proton return: The double-proton return in the third step of the LCM was a reverse process of the first step, and Ser144 was reprotonated by His257 (Figure 5), while interestingly, Ser144 protonated the newly formed oxyanion to finish this step (Figure 7). The double-proton transfer was precisely synchronous (as observed from Figure 7) and almost barrierless, with a free energy barrier of 1.6 kcal/mol. After the feedback of the proton, the 1,2-diacetin was far away from the catalytic triad (4.04 Å to 6.52 Å in Figure 7), which may be due to the presence of the water molecule at the catalytic active center.

1,2-diacetin to 1,3-diacetin: The last step to complete the 1,2-diacetin to 1,3-diacetin of the LCM was the opening of a five-membered ring at sn-2 C-O, accompanied by a transfer of the proton from the sn-2 carbonyl carbon to the sn-1 ester oxygen (Figure 2a). The corresponding TS4 and the production (P_LCM_) were optimized and are depicted in Figure 7. This step was the rate-limiting step with a highest activation energy barrier (33.3 kcal/mol), suggesting that the present simulation of LCM seemed to be impossible at room temperature, even if an exergonic reaction was obviously found (8.4 kcal/mol). Alternatively, as displayed in the pathway of Figure 2b, one water molecule was designed in the quantum cluster model (Figure 3) to mediate the transfer of the proton, because the aforementioned report verified that the participation of water could lower the activation energy barrier of the rate-limiting step in the NCM. Figure 7 depicts the optimized structure of TS4 with water. As observed in the NCM, the water molecule also acted as the shuttle of the proton to facilitate the transfer of the proton from the sn-2 carbonyl carbon to the sn-1 ester oxygen in the LCM by expanding the undesirable tetratomic ring to the favorable hexatomic ring. Compared to pathway a, the aid of water significantly lowered the activation energy barrier of this step from about 14.5 kcal/mol to 18.8 kcal/mol, which is surprisingly consistent with the value (17.8 kcal/mol) derived from the available experimental rate [31].

## 4. Conclusions

In the present work, we analyzed two different conceivable mechanisms (NCM and LCM) of AM during the process of LCSs by DFT, using the hybrid functional B3LYP calculations in the Gaussian 09 software package. For NCM, the stepwise pathway was more favorable than the concerted one due to the lower energy barrier (41.8 < 55.4 kcal/mol). With the aid of one water molecule, the energy barrier of the rate-limiting step in the stepwise pathway was further reduced to 31.7 kcal/mol, but it was still so insurmountable that the AM was extremely slow to occur at room temperature, according to Arrhenius’ equation. Alternatively, LCM, with the help of one water molecule, can significantly accelerate the rate of AM due to the minor free energy barrier at room temperature. Specifically, the LCM of AM could be divided into four elementary steps: (1) His257 deprotonated Ser144, while Ser144 captured the hydroxyl proton on the sn-1 of the substrate, 1,2-diacetin, forming a double-proton transfer process; (2) the oxyanion on the sn-1 of the 1,2-diacetin nucleophilic attack on the sn-2 carbonyl carbon formed a five-membered ring; (3) Ser144 was reprotonated by His257, while Ser144 protonated the carbonyl oxyanion of the substrate, 1,2-diacetin, to complete the return of the double proton; and (4) this was accompanied by a transfer of the proton from the sn-2 carbonyl carbon to the sn-1 ester oxygen, a five-membered ring was opened, involved with one water molecule, and thus 1,2-diacetin became 1,3-diacetin, completing the process of AM. Of all the steps, the last step was the rate-limiting step of the LCM, with a reaction energy barrier of 18.8 kcal/mol, which was more consistent with the value (17.8 kcal/mol) derived from the available experimental data. Interestingly, we also found that the catalytic triad (Asp-His-Ser) in lipase can be used as a generalized acid/base, and the added water molecule can be applied as a “proton shuttle” during the process of AM, to effectively reduce the required energy barrier in both the NCM and LCM. In brief, the present study provided a clear elucidation of the mechanism of AM, as it emerged during the process of LCSs. Generally, the energy barrier of the AM involved in LCM was about 2 times lower than that of the AM involved in NCM, suggesting that the presence of lipase could accelerate AM by reducing the energy barrier of the AM reaction.
